# In Vivo and In Vitro Evaluation of Pharmacological Potentials of Secondary Bioactive Metabolites of* Dalbergia candenatensis* Leaves

**DOI:** 10.1155/2017/5034827

**Published:** 2017-12-26

**Authors:** Md. Anisuzzman, Md. Mahedi Hasan, Amit Kumar Acharzo, Asish Kumar Das, Sinthia Rahman

**Affiliations:** Pharmacy Discipline, Life Science School, Khulna University, Khulna 9208, Bangladesh

## Abstract

*Background. Dalbergia* species has wide range of secondary metabolites and is traditionally used in treatment of painful micturition, swelling, and leprosy and as blood tonic. The study evaluates membrane stabilizing, anticoagulant, analgesic, cytotoxic, subacute anti-inflammatory, and depression potentials of* D. candenatensis *leaves metabolites.* Methods*. Membrane stabilizing activity was evaluated by hypotonic induced hemolysis assay, whereas anticoagulant activity is done through extrinsic pathway by measuring prothrombin time. Analgesic action, cytotoxic effect, and subacute anti-inflammatory activity were determined by acetic acid induced writhing model, brine shrimp lethality bioassay, and formaldehyde induced model, respectively. Depression activity was measured by the Open Field, Hole Cross, Hole Board, and thiopentone induced sleeping time measuring methods.* Results*.* D. candenatensis *contains phenolic, flavonoid, and tannin, quantified as 416.25 mg, 330.00 mg, and 432.22 mg Gallic Acid Equivalent/100 g of dry extract, respectively. Extract showed maximum inhibition of writhe, hemolysis, and edema, approximate to 57.14%, 36.62%, and 34.1%, respectively. LC_50_ value for nauplii was 151.499 *μ*g/ml. Mean prothrombin time was approximate to 31.0 ± 2.31 seconds at 1.0 mg/ml. Extract showed depression activity, and maximum sleeping time was noted to be about 141 minutes.* Conclusion*.* D. candenatensis *leaves show dose dependent membrane stabilizing, anticoagulant, depression, analgesic, moderate cytotoxic, and subacute anti-inflammatory activities.

## 1. Introduction

From time immemorial, men have used several natural sources to combat with diseases rather than concede the fate of diseases [1]. Among many natural sources, men have been using plants mostly because of its availability and routinely used as foods or other purposes. Moreover, it is believed that natural product derived from plants may have fewer side effects to life [2]. The perception of therapeutic use of plants has continued with the development of human civilization and knowledge. Scientists have been trying hard to isolate different chemical compounds from plants to identify therapeutically active compounds based on various biological and pharmacological studies. Today, almost 33% of the drugs produced in the developed countries are derived from plants [3]. In view of this, the present studies were designed for preliminary screening of phytochemicals and evaluation of membrane stabilizing, anticoagulant, analgesic, subacute anti-inflammatory, depression, and cytotoxic activities of leaves of* Dalbergia candenatensis *Prain*. D. candenatensis *is a small- to medium-size tree which belongs to Fabaceae family (alternatively known as the Leguminosae). Geographically, the plant widely scattered throughout the world including mangrove forest of Bangladesh [4, 5].* D. candenatensis* is commonly known as Chanda Lota [4]. Various species of* Dalbergia *are traditionally used in the treatment of different ailments like analgesic, anti-inflammatory, antimicrobial, cough, haemorrhages, and leprosy [5]. The wood of* D. candenatensis* is used as a blood tonic, expectorant, antifungal, and antibacterial substances [6]. The CHCl_3_ and MeOH extracts of heartwood were reportedly found to display antibacterial, antifungal activity, and cytotoxic activity against the P-388 lymphocytic leukemia test system in vitro [7]. The CHCl_3_ and MeOH extracts of heartwood have been reported to possess several types of flavonoids like mucronulatol, claussequinone, 5-hydroxybowdichione, formononetin, and vestitol [7]. The CH_2_Cl_2_ extract of the heartwood of* D. candenatensis *has been also reported to possess Candenatenin A, Candenatenin B, Candenatenin C, Candenatenin D, Candenatenin E, Candenatenin F, 3,5-dihydroxy-7-methoxyflavanone, 4-hydroxy-3-methoxy-8,9-methylenedioxypterocarpan, nutiducol, and sophoraflavanone with cytotoxic activity [6]. The acetone extract of air-dried heartwood of* D. candenatensis* was also investigated to obtain Candenatenin G, Candenatenin H, Candenatenin I, Candenatenin J, Candenatenin K, dinklagin A, stipulin, (R)-4-methoxydalbergione, and melilotocarpan A [6]. The methanol extract of wood of D. candenatensis exhibited significant estrogenic activity on ER*β* [8]. Previous records of phytochemical and biological studies of* D. candenatensis* except on its wood are scanty. Therefore, present study was concerned with the investigation of membranes stabilizing, anticoagulant, analgesic, subacute anti-inflammatory, neuropharmacological, and cytotoxic activity of leaves of* Dalbergia candenatensis* Prain.

## 2. Materials and Methods

### 2.1. Plant Collection and Extraction

The leaves of* Dalbergia candenatensis* were carefully collected from Sundarban, Bangladesh, during the month of January, 2016, at daytime. After collection, the sample was identified by the authorities of Bangladesh National Herbarium, Mirpur, Dhaka, where a voucher specimen (DACB Accession Number 36783) was deposited for further reference. The leaves were washed with fresh water, cut into small pieces, and then shred-dried for up to fourteen days. The dried leaves were grounded into fine powder by means of Capacitor Start Motor, China. The crude extract was obtained by cold extraction method by taking 260 g powders in 800 ml of 98% ethanol into a clean and air-tight glass vessel for fourteen days at room temperature with occasional shaking and stirring. After aforementioned maceration, mixture was filtered through Whatman filter paper to separate the extract from the plant debris. The extract was concentrated initially by rotary evaporator at reduced pressure and finally by open air. The yield was found to be 5.77% w/w.

### 2.2. Chemicals and Drugs

Diclofenac sodium, Indomethacin, thiopental sodium, and diazepam were collected from Square Pharmaceuticals Ltd., Bangladesh. Warfarin was collected from Incepta Pharmaceuticals Ltd. Vincristine sulphate was collected from Beacon Pharmaceuticals Ltd. All other analytical grade chemicals were purchased from Sigma Chemicals, USA.

### 2.3. Test Animals

Young healthy Swiss-albino mice aged 4-5 weeks and rats aged 3-4 months were purchased from The International Centre for Diarrheal Disease Research, Bangladesh. They were kept for seven days in the animal house of the Pharmacy Discipline, Khulna University, Bangladesh, for adaptation. The animal house was maintained with relative humidity 55%–65%, room temperature 25 ± 2°C, and 12/12 h light/dark cycle. ICDDR B-formulated diet and purified water were fed to the animals. Brine shrimp nauplii hatched in the laboratory. These animals were treated by following the “Ethical Principles and Guidelines for Scientific Experiments on Animals (1995)” instructed by the Swiss Academy of Medical Sciences and the Swiss Academy of Sciences for the entire experiment period.

### 2.4. Phytochemical Screening

Phytochemical screening of ethanol extract of leaves of* Dalbergia candenatensis* was carried out according to standard quantitative procedures [9]. The crude extract was investigated to probe alkaloid, phenolic, steroid, reducing sugar, saponin, tannin, and flavonoids.

### 2.5. Evaluation of Membrane Stabilizing Activity

Membrane stabilizing activity of* D. candenatensis* was measured by hypotonic induced haemolysis assay [10, 11]. Fresh blood sample was collected into centrifuge tube containing trisodium citrate (3.8% w/v) and centrifuged at 3,000 rpm for 10 minutes. Then, supernatant was carefully removed by using pipette. The packed erythrocytes were resuspended in fresh 0.9% sodium chloride and mixed thoroughly followed by centrifugation again for 5 minutes to remove the supernatant as aforementioned. This process continued until clear supernatant was obtained. Then 2% (v/v) erythrocyte suspension was prepared by using packed red blood cells with 0.9% sodium chloride. During experiment, 0.5 ml stock erythrocyte suspension was mixed with 5 ml hypotonic buffer saline (50 mMols sodium chloride in 10 mMols sodium phosphate, pH = 7.4) into four test tubes and then 1 ml crude extract with concentrations of 0.25, 0.50, 1.0, and 2.0 mg/ml added, respectively. Standard sample was prepared with the mixture of 1 ml Indomethacin of 0.10 mg/ml, 0.5 ml stock erythrocyte suspension, and 5 ml hypotonic buffer saline, while the control sample contains only the same volume of distilled water rather than test sample or standard drug. Then, test tubes were incubated for 10 minutes at room temperature. After incubation it was subjected to centrifugation for 10 minutes at 3,000 rpm. Finally, the supernatant was collected and absorbance was measured at 540 nm.

### 2.6. Evaluation of Anticoagulant Activity

Anticoagulant activity of* D. candenatensis* was measured by using prothrombin time test method [12]. Blood samples were collected from healthy volunteers aged 20–25 to accomplish the experiment. Disposable syringes were used to withdraw blood from volunteer. The blood was taken into centrifuge tubes that previously contained 3.8% trisodium citrate to inhibit blood coagulation during transportation. The centrifuge test tube was centrifuged at 3,000 rpm for 15 min to obtain pure platelet plasma. Then the plasma was carefully separated from suspended blood particle and stored at 4°C until use. Several concentrations (0.25, 0.50, 1.00, 2.00, and 4.00 mg/ml) of* D. candenatensis* leaves extracts, 0.2 ml test plasma of each individual, and 0.3 ml CaCl_2_ (25 mMols) were mixed together into a test tube. Then 0.1 ml of 0.9% saline, 0.2 ml test plasma of each individual, and 0.3 ml CaCl_2_ (25 mMols) were mixed together into another test tube for negative control whereas 0.1 ml warfarin taken in place of the extract, 0.2 mL test plasma of each individual, and 0.3 ml CaCl_2_ (25 mMols) were mixed together into another test tube for positive control. Each tube was incubated at 37°C and the clotting of blood samples was inspected visually by tilting the test tube frequently. Each of the tests was carried out 5 times with the clotting time recorded using stopwatch.

### 2.7. Evaluation of Acute Toxicity

This was performed to determine the safe dose(s) to be used in different tests. The mice were kept in fasting condition for 16 h. The mice were divided into 5 groups each containing 6 mice and the extract was orally administered at the doses of 500, 1,000  1,600, and 2,000 mg/kg body weight, while the control group received distilled water. The animals were kept 72 h for observation of death of mice. General signs and symptoms of toxicity were noted for each group according to OECD guide with slight modification [13, 14].

### 2.8. Evaluation of Analgesic Activity

The analgesic activity was evaluated by acetic acid induced writhing model in mice [15]. The experimental animals were randomly divided into five groups having five mice each. The mice of each group were accurately weighed and each experimental group was properly marked. Negative control group received only 1% (v/v) Tween-80, whereas standard group received standard drug, diclofenac sodium at a dose of 25 mg/kg body weight as oral suspension. In test groups (I, II, and III), 100, 250, and 500 mg/kg body weight extracts doses were administered. All doses were given orally with the help of sterile feeding needle. After 30 minutes, (0.7% v/v) acetic acid solution was administered through intraperitoneal injection (IP injection) to all groups. The mice were rested for few minutes because of better absorption of acetic acid. After 5 minutes, the number of writhings was counted for 15 min. The incomplete writhing was taken as half-writhing, so two half-writhings were taken as full writhing. The number of writhings in the control was supposed to be 100% and % inhibition was calculated as follows:

(1) % Inhibition of writhing=100−treated meancontrol mean×100.

### 2.9. Evaluation of Cytotoxic Activity

Cytotoxic activity of ethanol extract of leaves of* D. candenatensis *was exploited with brine shrimp lethality bioassay [16]. A total of 38 g of sea salt was weighed accurately and dissolved in distilled water to make simulated seawater. Seawater was taken into small tank and eggs were poured into it. The small tank was kept at 28°C in front of a lamp for two days to incubate the shrimp eggs and to be matured as nauplii. During the experiment, the extracts were dissolved in simulated seawater by using DMSO and serial dilution was performed to obtain solutions of varying concentrations—1, 2, 4, 8, 16, 32, 64, 128, 256 and 512 *μ*g/ml. Serial dilution was also performed to obtain varying concentrations of standard drug, Vincristine sulphate (0.3125, 0.625, 1.25. 2.50 and 5.00 *μ*g/ml) by using simulated seawater. Then extract or standard drug in different desire concentrations were then added to the premarked test tube containing 10 live brine shrimp nauplii in 10 ml simulated seawater. Simulated seawater with DMSO served as negative control. The test tubes were left uncovered under the lamp and the number of surviving shrimps was counted after 24 hours with the help of magnifying glass and the results were noted. The test was performed in triplicate to avoid statistical error. From this, the percentage of lethality of brine shrimp nauplii was calculated at each concentration following the equation

(2) $$\begin{array}{c} \%\text{ Mortality}=\left[\;\frac{\left(\text{Avg. no. of alive shrimp of control}-\text{Avg. no. of alive shrimp of sample}\right)}{\text{Avg. no. of alive shrimp of control}}\;\right]\times 100. \end{array}$$

### 2.10. Evaluation of Subacute Anti-Inflammatory Activity

Formaldehyde induced subacute inflammation model was implemented to evaluate the anti-inflammatory activity of ethanol extract of* D. candenatensis *leaves with slide modification [17]. In this experiment, 25 rats were randomly selected and classified into five different groups termed as negative control group, positive control group, and test groups (I, II, and III). Negative control group received only 1% (v/v) Tween-80 at a dose of 10 ml/kg body weight, while positive group received standard drug, Indomethacin at a dose of 10 mg/kg body weight as oral suspension. In the test groups (I, II, and III), extracts were administered at 100, 250, and 500 mg/kg body weight dose. All doses were given orally with the help of sterile feeding needle. After thirty minutes, the subacute inflammation was induced in all groups by subcutaneous injection of 0.1 ml of 2% formaldehyde in the right paw of each rat. The linear circumference of the injected paw was measured at 1 h, 2 h, 3 h, 4 h, 24 h, and 48 h after formaldehyde injection.

The percentage inhibition of edema was calculated as per the following equation:

(3) $$\begin{array}{c} \%\text{ Inhibition of edema}=100\times \left(\;l_{0}-\frac{l_{1}}{l_{0}}\;\right), \end{array}$$

where *l*_0_ is change in paw circumference in control group and *l*_1_ is change in paw circumference in drug treated group or test group.

### 2.11. Evaluation of Neuropharmacological Activity

#### 2.11.1. Thiopental Sodium Induced Sleeping Time

In these experiments, 25 experimental laboratory mice were arbitrarily chosen and accurately weighed. The mice were divided into five groups termed as negative control group, positive control group, and test groups (I, II, and III). Five mice in each group were kept in five cages separately. Each mouse of negative control group received orally 1% (v/v) Tween-80 in distilled water at the dose of 10 ml/kg body weight. The mice of positive control group received standard drug diazepam at the dose of 3 mg/kg body weight. The mice of each test group (I, II, and III) received crude extract at the doses of 100, 250, and 500 mg/kg body weight. All doses were given orally with the help of feeding needle. After 30 minutes, thiopental sodium (40 mg/kg) was given intraperitoneally to all groups for inducing sleep. The onset time of sleep was noted for all the animals. After induction of sleep, mice were placed in the inverted position and when sedation was over, the mice came to normal posture, and time was noted. The interval between loss and recovery of righting reflex was used as index of hypnotic effect. The time interval between injection of thiopental sodium and start of sleep was recorded as latency time [18].

#### 2.11.2. Open Field Test

In this experiment, 25 mice were arbitrary chosen and divided into five different groups termed as negative control group, positive control group, and test groups (I, II, and III). Negative control group received only 1% (v/v) Tween-80 at a dose of 10 ml/kg body weight, while positive group received standard drug, diazepam at a dose of 3 mg/kg body weight as oral suspension. The test groups (I, II, and III) were treated with suspension of plant extracts at the oral dose of 100, 250, and 500 mg/kg body weight. All doses were given orally with the help of sterile feeding. After respective treatment, animals were placed individually in one of the corners of square grids (100 cm × 100 cm × 40 cm). The number of squares traveled by the mice was monitored for 3 min at 0, 30, 60, 90, and 120 minutes during the observation period. During the experiment silent environment was strongly maintained [19].

#### 2.11.3. Hole Board Test

Mice were divided into 5 groups and each group comprised 5 mice with 20–25 g in weight. Group I was given 1% Tween-80, Group II was treated with diazepam at 3 mg/kg body weight dose, and Groups III, IV, and V termed as test groups were given ethanol extract of* D. candenatensis* at the doses of 100, 250, and 500 mg/kg body weight, respectively. At the beginning of the test, mouse was placed in the edge of the board. The number of head dips into the holes was counted as the measurement for a period of 3 minutes on 0, 30, 60, 90, and 120 minutes for the entire observation period. The experiment was carried out in a sound attenuated room [20].

#### 2.11.4. Hole Cross Test

After respective treatment of aforementioned group, mice were placed individually in the darker chamber of the box, segregated by a wall with hole into dark and white chambers. The total number of crosses through the hole from one chamber to the other by the mouse of each group within 3 minutes was counted on 0, 30, 60, 90, and 120 minutes. The experiment was conducted in a sound attenuated room [21].

### 2.12. Data Analysis

“*t*” test was applied for unpaired data and comparing more than two samples, the analysis of variance (ANOVA) test was applied. The LC_50_ value of brine shrimp lethality bioassay was calculated with the help of probit analysis software (Ldp line software, USA). Data obtained from this study were expressed as mean ± SEM. *p* values less than 0.05 were statistically significant.

## 3. Results

### 3.1. Phytochemical Screening

The crude ethanol extract of* D. candenatensis *was subjected for chemical group tests and revealed the presence of reducing sugar, tannin, phenolic, flavonoid, steroid, alkaloid, glycoside, and protein and absence of saponin. Extract was quantified as 416.25, 330.00, and 432.22 mg Gallic Acid Equivalent/100 g dry extract of phenolic, flavonoid, and tannin content, respectively.

### 3.2. Membrane Stabilizing Activity

Ethanol extract of* D. candenatensis *leaves exhibited dose dependent inhibition of hemolysis. Compared to control, maximum inhibition was found at 2.0 mg/ml and lowest inhibition was found at 0.25 mg/ml (Table 1).

### 3.3. Evaluation of Anticoagulant Activity

In prothrombin time test, different concentration of extracts produced increased clotting times in a dose dependent manner. For evaluation of anticoagulant activity, at 2.0 mg/ml and 4.0 mg/ml doses, the mean prothrombin time of blood was measured at 67 and 176 seconds, respectively, which were comparable to warfarin at 0.1 mg/ml (Table 2).

### 3.4. Evaluation of Analgesic Activity

The crude extract exhibited 23.47%, 40.82%, and 57.14% writhing inhibition in mice at oral doses of 100, 250, and 500 mg/kg body weights of mice, respectively. On the other hand, the standard drug Diclofenac sodium exhibited inhibition of 76.53% at 25 mg/kg body weight dose (Table 3).

### 3.5. Evaluation of Cytotoxic Activity

**Figure 1:**
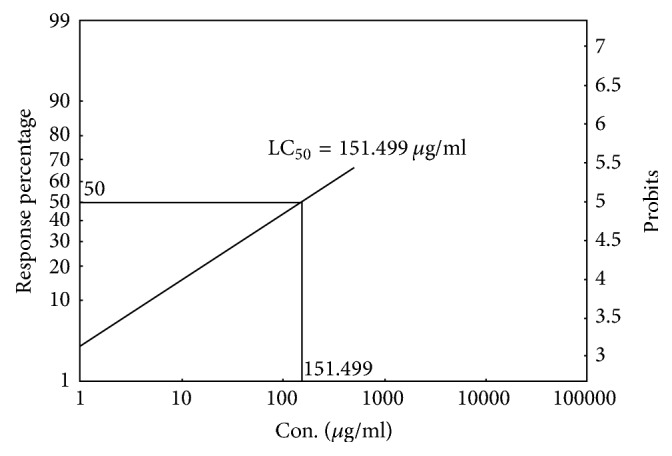
Cytotoxic effect of ethanolic leaves extract of* D. candenatensis*.

**Figure 2:**
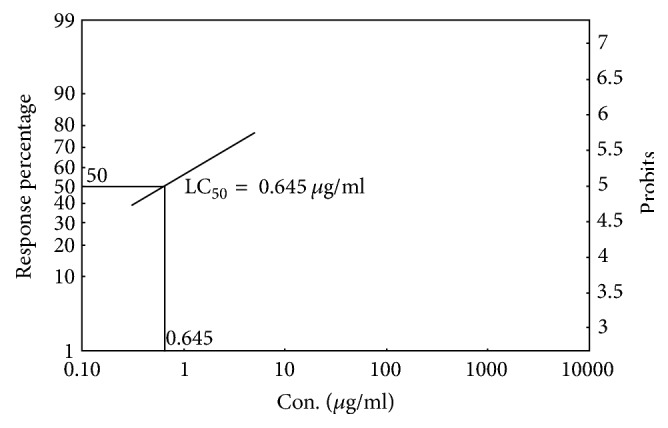
Cytotoxic effect of ethanolic solution of standard (Vincristine sulphate).

The mortality rate of brine shrimp was increased proportionally with increased concentration of the sample. An approximate linear correlation obtained by plotting percent mortality versus log concentration on the graph paper. The crude extract and standard (Vincristine sulphate) showed 50% mortality (LC_50_) of brine shrimp nauplii at concentration of 151.499 *μ*g/ml (Figure 1) and 0.645 *μ*g/ml, respectively (Figure 2).

Supplementary data associated with this figure is available here.

### 3.6. Evaluation of Anti-Inflammatory Activity

The swelling induced by formaldehyde was significantly (*p* < 0.05) reduced by the crude ethanol extract in a dose dependent manner. The extract exhibited highest reduction of the size of the edema by 34.10% at 500 mg/kg body weight of rats paw in comparison with Indomethacin that reduced the size of the edema by 51.4% at 10 mg/kg body weights of rat after 48 hours (Table 4).

### 3.7. Evaluation of Neuropharmacological Activity

#### 3.7.1. Evaluation of Thiopental Sodium Induced Sleeping Time

The ethanol extracts of* D. candenatensis* statistically significantly reduced the time for the onset of sleep and increased the duration of sleep as compared to the control in dose dependent manner. The maximum duration of sleep time was observed to be 141 minutes at 500 mg/kg body weight dose (Table 5).

#### 3.7.2. Open Field Method

The crude extracts displayed statistically significant reduction in the movements in mice as compared to control. The decrease in the movement was manifested at 2nd observation persistent until 4th observation at every tested dose (100, 250, and 500 mg/kg). Diazepam exhibited similar results but the effect was fairly stronger than the extracts (Table 6).

#### 3.7.3. Hole Cross Method

The crude extracts displayed statistically significant reduction of locomotors activity in mice at every tested dose (100, 250, and 500 mg/kg) compared to control. The decrease in the locomotors activity was manifested at 2nd observation persistent until 4th observation. Diazepam (positive control) exhibited similar results but the effect was fairly stronger than the extracts (Table 7).

#### 3.7.4. Hole Board Method

In the Hole Board Test, the crude extract at each dose showed significant reduction in the number of head dips compared to control, although the effect of diazepam was strong than that of the results of the crude extracts. The effect was started from 2nd observation of the experiment and lasted to 4th observation (Table 8).

### 3.8. Acute Toxicity Test

In oral acute toxicity test, the highest dose (2,000 mg/kg body weight of mice) of the leaves extract did not show any mortality and side effect.

## 4. Discussion

Our preliminary phytochemical screening confirmed the presence of secondary bioactive metabolites such as alkaloids, glycosides, flavonoids, steroids, and tannins in the ethanolic extract of leaves of* Dalbergia candenatensis. *The present study investigated the membrane stabilizing, anticoagulant, analgesic, cytotoxic, anti-inflammatory, and neuropharmacological properties of* D. candenatensis *secondary bioactive metabolites. Acute oral administration of ethanolic extract at the higher dose of 2,000 mg/kg did not display any allergic manifestations or mortality during the observation period of 72 hours after administration. Therefore, it is conceivable that* D. candenatensis* leaves extract may not be toxic at our experimental higher doses up to 2,000 mg/kg and thus, it ensures good range of therapeutic index.

Erythrocytes lysis means the disruption of integrity of cell membrane. Tissue damage is prevented through inhibition of lysosome stored hydrolytic enzymes release [22]. Phytochemical screening of* D. candenatensis *revealed that plant extracts were rich in flavonoid and tannin which was associated with membrane stabilizing effect because it inhibits the release of lysosomal content of neutrophils at the site of inflammation where erythrocyte membrane may be simulated to the lysosomal membrane and allowed significant protection of the erythrocyte against lysis induced by hypotonic solution [23]. Absence of saponin in plant extracts also prevents blood cell lysis [24]. The highest inhibition was found to be about 36.62%, while the lowest inhibition was approximate to 8.45% at 0.25 mg/ml and 2.0 mg/ml extract dose, respectively.* D. candenatensis* has exhibited outstanding membrane stabilizing activity.

Prothrombin time test measures how long blood takes to clot. In prothrombin time (PT) test, fibrin clot formation in response to tissue injury is the most clinically relevant event of hemostasis under normal physiological conditions. This process is the result of the activation of the extrinsic pathway. So, the agent prolonging the prothrombin time by interfering with blood coagulation pathway is referred to as anticoagulation agent [25]. The prothrombin test specifically evaluates the presence of factors VII, V, and X, prothrombin, and fibrinogen. A prothrombin time within the 7–15-second ranges indicates that the patient has normal amounts of the above clotting factors. A prolonged prothrombin time indicates a deficiency in any of factors VII, X, and V, prothrombin, or fibrinogen. It means that the patient may have a vitamin K deficiency (vitamin K is a cofactor in the synthesis of functional factors II, VII, IX, X, and XIII and prothrombin) or liver disease which is attributed to production of plasma protein factors [26]. Extracts containing alkaloids and flavonoids may be acting on the extrinsic cascade of clotting by binding with factors resulting in complex formations which inhibit the conversion of prothrombin to thrombin and finally inhibiting the conversion of the soluble fibrinogen to insoluble fibrin clot [27, 28]. At 2.0 mg/ml and 4.0 mg/ml doses, the mean prothrombin time of blood was measured at 67 and 176 seconds, respectively, which were comparable to warfarin at 0.1 mg/ml dose. Thus, the study showed that* D. candenatensis* has exhibited remarkable anticoagulant activity.

Acetic acid induced abdominal contractions cause writhing peripherally in mice within very short time duration. Intraperitoneal administration of acetic acid (0.7%) causes localized inflammation through the release of endogenous pain mediators where stimulus causes the release of free arachidonic acid from tissue phospholipids due to the action of phospholipase A2 and acyl hydrolases. Prostaglandin, thromboxane, and prostacyclin are synthesized via the cyclooxygenase pathway from arachidonic acid [29]. The released prostaglandins, mainly prostacyclin (PGI2) and prostaglandin E, are responsible for sensation of local pain [30]. Substances that lower the number of writhings in mice demonstrate analgesia by inhibition of prostaglandin synthesis, a peripheral mechanism of pain inhibition. Phenolic and different flavonoids like rutin, quercetin, and luteolin are responsible for analgesic properties [31, 32]. Ethanol extract of* D. candenatensis* considerably lowered the number of writhings in mice with dose dependent manner possibly due to the presence of phenolic and flavonoids agents. The standard drug diclofenac sodium inhibits 76.53% writhing at a dose of 25 mg/kg body weight whereas 57.14% writing inhibition was found at 500 mg/kg dose of extracts just after 15 minutes of the administration of acetic acid pain inducer (Table 3).

The general toxicity of the crude extract was measured by means of a rapid, simple, and convenient technique using brine shrimp lethality bioassay. Though brine shrimp lethality bioassay does not appertain to any specific pharmacological activity, it provides a reasonably well correlation between cytotoxicity and anticancer activities [33]. Anticancer agent shows maximum level of toxicity in this assay. However, any agent that shows toxicity in this assay might not be an anticancer agent but might be an antimalarial agent or insecticidal agent or molluscicidal agent or larvicidal agent [33, 34]. The sample extracts LC_50_ was 151.499 *μ*g/ml (Figure 1) and Vincristine sulphate LC_50_ was 0.645 *μ*g/ml (Figure 2) which showed low level of general toxicity of extract in comparison with standard drug. It has been previously reported that LC_50_ value above 250 *μ*g/ml indicates weak toxic potentiality [35]. This low level of toxicity might be attributed to the presence of plants flavonoids, tannins, and alkaloids type bioactive metabolite and absence of saponin [36]. Thus, the test extract possibly assured its safety in this assay in relation to its use.

It is well known that inhibition of edema induced by formalin in rats is one of the most suitable test procedures to screen antiarthritic and anti-inflammatory agents, as it closely resembles human arthritis [37]. Injection of formalin causes an immediate and intense spontaneous biphasic nociceptive response through the release of several inflammatory mediators including prostaglandin, prostacyclin, and leukotriene [38]. Early phase is caused by a direct effect of formalin on nociceptors whereas the late phase shows tonic response due to activation of neurons of dorsal horns of the spinal cord [38]. In vivo anti-inflammatory activity of flavonoid and flavone derivatives is via modulation of proinflammatory gene expression, for example, inducible NOS and COX-2.* D. candenatensis *extract showed maximum percentage of inhibition at 3-4 hours after administration of formalin and then gradually reduces the percentage of paw edema. This test was carried out for 48 hours. Higher dose (500 mg/kg) showed mainly the highest percentage of inhibition (36.71%–36.84%) of rats paw edema value. Preliminary phytochemical screening of ethanolic* D. candenatensis *leaves extract revealed the presence of flavonoids which may be responsible for the observed subacute anti-inflammatory effect [39]. Hence, it is suggested that ethanolic extract of leaves may provide benefits in the management of arthritis by inhibition of paw edema. This is in accordance with the findings reported in the related literature, which have shown intense edema after three hours, mild edema within three days, and no edema at day seven [40].

Depression effect carried out by In Vivo Hypnosis, Open Field, Hole Board, and Hole Cross methods. The Open Field test measures a number of facets of behavior beyond simple locomotion. The Hole Board Test (HBT) is an experimental method used in scientific research to measure anxiety, stress, neophilia, and emotionality in animals. Because of its ability to measure multiple behaviors, it is a popular test in behavioral pharmacology but the results are controversial. Moreover, the validation of anxiety was carried out by measuring external signs, through Hole Cross tests. There are several reports which demonstrated that the alkaloids, glycosides, and flavonoids rich plant extracts possess sedative, anxiolytic, and antiepileptic properties mediated through their affinity with benzodiazepine site of GABAergic complex system or are direct or indirect modulators of this receptor's increases in GABA activity in the brain producing drowsiness and facilitating or maintaining sleep [41–45]. Besides, nonspecific CNS depression can also be attributed by tannin [46]. Therefore it appears that the above-mentioned phytochemicals present in the* D. candenatensis *leaves extract may contribute at least in part to the sedative and hypnotic effects on the CNS. We started our investigation to evaluate CNS depression effects of* D. candenatensis *leaves extract by recording spontaneous locomotors activity of mice in Hole Cross and Open Field tests. In these tests, any agents with sedative activity will cause reduction in the number of movements and interruption in curiosity of the new environment [47]. Our result displayed that the oral administration of test extract at the doses 100, 250, and 500 mg/kg caused a marked reduction in number of holes crossed and lethargy to new environment which was reverse for CNS stimulating agent. The suppression effect was found at 30 min and continued up to 120 min after administration of leaf extract. All tested doses produced significant inhibition of locomotion. The suppression of locomotors by inducing* D. candenatensis *crude extract referred its potentiality to depress central nerves system [47]. Another important observation was achieved in the Hole Board Test. This test is well-established as a means to assay potential anxiolytic and sedative effects of any agents by observing the exploratory behavior in rodents. Head-dipping behavior of the animals is directly related to their emotional state [48]. Based on this observation, it was suggested that the expression of an anxiolytic state in animals might be reflected by an increase in head-dipping behavior [48] while a decrease in the number of head dips was found to be correlated with the depressant effect [49, 50]. Our results revealed that the ethanolic extract of* D. candenatensis *caused a dose dependent reduction in head-dip response in the animals from 2nd observation of the experiment and lasted to 4th observation, suggesting that the extract possesses sedative activity rather than anxiolytic potentials [47].

Our above findings were further supported by the results observed in thiopental sodium induced sleeping time determination test. This test is a classical method in behavioral pharmacology to investigate the sedative and hypnotic properties. In our study, the acute oral treatment with different doses of* D. candenatensis *extract significantly modified the latency to induce sleep as well as increasing duration of hypnosis induced by thiopental sodium (maximum sleeping time was noted to be 141 minutes at 500 mg/kg body weight). As expected, similar types of effects were observed by the administration with diazepam at 1 mg/kg dose. Substantial evidence revealed that the CNS depressant barbiturates, such as thiopental sodium, bind to the barbiturate binding site on the GABA_A_ receptor complex and potentiate GABA-mediated hyperpolarization of postsynaptic neurons [41]. Our results suggest that there might be a relationship between the sedative effect produced by experimental leaves extract and the sedation inductive capacity of diazepam. Therefore, it is possible that the GABAergic system may participate in the* D. candenatensis *leaves extract-induced enhancement of the effects of thiopental sodium.

## 5. Conclusion

Crude extracts of* Dalbergia candenatensis *were subjected to phytochemical and pharmacological investigations to validate the traditional use of it.* D. candenatensis *showed wide range of potential source of secondary bioactive metabolites that exhibited membrane stabilizing, analgesic, anticoagulant, sedative-hypnotic, subacute inflammatory, and cytotoxic activities. The effect is rapid, long-lasting, and statistically significant at all the experimental doses tested. However, further studies are needed to isolate bioactive compound(s) and elucidate the precise molecular mechanisms in order to establish the safe and effective dosage and, additionally, verify the possibility of its use in the prevention and cure of diseases, contributing to improving the health of the population through increased access to herbal remedies and medicinal plants and their rational use for the pharmacological activities of the experimental plant.

## Figures and Tables

**Table 1:** Membrane stabilising activity of *D. candenatensis *on hypotonic induced haemolysis (mean ± SEM, *n* = 5).

Groups	Dose	Optical density (OD)	% inhibition of haemolysis
Negative control (distilled water)	-	0.71 ± 0.003	-
Indomethacin (standard drug)	0.1 mg/ml	0.34 ± 0.006^*∗∗∗*^	52.11
Extract I	0.25 mg/ml	0.65 ± 0.003^*∗∗∗*^	8.45
Extract II	0.50 mg/ml	0.60 ± 0.003^*∗∗∗*^	8.45
Extract III	1.0 mg/ml	0.53 ± 0.003^*∗∗∗*^	25.35
Extract IV	2.0 mg/ml	0.45 ± 0.003^*∗∗∗*^	36.62

^*∗∗∗*^ *p* < 0.001, significant compared to control.

**Table 2:** Anticoagulant activity of *D. candenatensis* on prothrombin time (PT) of normal human plasma (mean ± SEM, *n* = 5).

Sample	Concentration of sample	Mean prothrombin time (sec)
Control	0.9% sodium chloride	7.0 ± 1.15

Warfarin sodium	0.1 mg/ml	122.0 ± 2.52^*∗∗∗*^

Ethanolic leaves extract of *D. candenatensis*	0.25 mg/ml	10.67 ± 1.45
	0.50 mg/ml	20.33 ± 2.40^*∗∗*^
	1.0 mg/ml	31.0 ± 2.31^*∗∗∗*^
	2.0 mg/ml	67.23 ± 2.71^*∗∗∗*^
	4.0 mg/ml	176.04 ± 2.41^*∗∗∗*^

^*∗∗*^ *p* < 0.01 and ^*∗∗∗*^*p* < 0.001, significant compared to control.

**Table 3:** Analgesic activity of *D. candenatensis* on acetic acid-induced writhing in mice (mean ± SEM, *n* = 5). See *supplementary data associated with this table. *

Animal groups	Dose	Mean writhing	% writhing	% inhibition of writhing
Control (1% v/v Tween-80 water)	10 ml/kg	19.60 ± 1.81	100	0
Diclofenac sodium	25 mg/kg	4.60 ± 0.68^*∗*^	23.47	76.53
Extract I	100 mg/kg	15.00 ± 0.71^*∗*^	76.53	23.47
Extract II	250 mg/kg	11.60 ± 1.08^*∗*^	59.18	40.82
Extract III	500 mg/kg	8.4 ± 1.33^*∗*^	42.86	57.14

^*∗*^ *p* < 0.05, significant compared to control.

**Table 4:** Anti-inflammatory activity of *D. candenatensis* on formaldehyde-induced subacute inflammation in rat (mean ± SEM, *n* = 5). See *supplementary data associated with this table*.

Groups	Dose (mg/kg)	Mean difference in paw diameter (% inhibition)					
		1 hr	2 hr	3 hr	4 hr	24 hr	48 hr
Control (1% v/v Tween-80 water)	10 ml/kg	1.10 ± 0.0071	1.25 ± 0.006	1.28 ± 0.004	1.33 ± 0.011	1.30 ± 0.014	1.26 ± 0.011
Indomethacin	10 mg/kg	0.82 ± 0.007^*∗∗∗*^ (24.4%)	0.85 ± 0.004^*∗∗∗*^ (32%)	0.45 ± 0.008^*∗∗∗*^ (64.84%)	0.51 ± 0.007^*∗∗∗*^ (61.65%)	0.57 ± 0.009^*∗∗∗*^ (56.15%)	0.61 ± 0.01^*∗∗∗*^ (51.5%)
Extract I	100 mg/kg	1.05 ± 0.007^*∗∗∗*^ (4.5%)	1.18 ± 0.007^*∗∗∗*^ (5.6%)	1.04 ± 0.01^*∗∗∗*^ (18.75%)	1.11 ± 0.007^*∗∗∗*^ (16.54%)	1.08 ± 0.009^*∗∗∗*^ (16.92%)	1.08 ± 0.008^*∗∗∗*^ (14.2)
Extract II	250 mg/kg	1.01 ± 0.011^*∗∗∗*^ (8.18%)	1.11 ± 0.007^*∗∗∗*^ (11.2%)	0.92 ± 0.009^*∗∗∗*^ (28.12%)	0.98 ± 0.007^*∗∗∗*^ (26.71%)	0.97 ± 0.08^*∗∗∗*^ (25.38%)	0.95 ± 0.01^*∗∗∗*^ (24.6%)
Extract II	500 mg/kg	0.97 ± 0.007^*∗∗∗*^ (11.8%)	1.08 ± 0.006^*∗∗∗*^ (13.6%)	0.81 ± 0.005^*∗∗∗*^ (36.71%)	0.84 ± 0.011^*∗∗∗*^ (36.84%)	0.84 ± 0.005^*∗∗∗*^ (35.38%)	0.82 ± 0.009^*∗∗∗*^ (34.9%)

*∗∗∗* indicates *p* < 0.001 when compared with control.

**Table 5:** Effect of *D. candenatensis* on thiopental sodium induced sleeping time in mice (mean ± SEM, *n* = 5).

Treatment	Dose (mg/kg)	Onset of sleep (min)	Duration of sleep (min)
Control (1% v/v Tween-80 water)	10 ml/kg	1.94 ± 0.1	61.60 ± 3.87
Diazepam	3 mg/kg	0.81 ± 0.06	152.60 ± 4.40^*∗∗∗*^
Test I	100 mg/kg	1.75 ± 0.06	90.40 ± 3.40^*∗∗∗*^
Test II	250 mg/kg	1.59 ± 0.12	113.20 ± 3.17^*∗∗∗*^
Test III	500 mg/kg	1.30 ± 0.07	140.60 ± 3.19^*∗∗∗*^

*∗∗∗* indicates *p* < 0.001 when compared with control.

**Table 6:** Effect of *D. candenatensis* on Open Field test in mice (mean ± SEM, *n* = 5).

Group	Number of squares crossed by the mice				
	0 min	30 min	60 min	90 min	120 min
Negative control	37.5 ± 3.5	35.5 ± 2.22	35.5 ± 3.71	36.5 ± 3.28	33.25 ± 3.71
Positive control	35.5 ± 2.5	18.5 ± 1.32^*∗∗*^	15.0 ± 1.08^*∗∗∗*^	13.5 ± 1.85^*∗∗∗*^	13.25 ± 1.93^*∗∗∗*^
Test I (100 mg/kg)	35.25 ± 4.57	30.75 ± 4.03	29.25 ± 4.80	27.27 ± 2.46	29.25 ± 4.83
Test II (250 mg/kg)	36.25 ± 2.95	30.75 ± 2.92	26.0 ± 3.08	25.5 ± 2.46	26.0 ± 3.39
Test III (500 mg/kg)	34.5 ± 2.25	27.0 ± 3.03	22.5 ± 2.50^*∗*^	20.0 ± 3.85^*∗∗*^	19.25 ± 3.33^*∗*^

*∗* indicates *p* < 0.05, *∗∗* indicates *p* < 0.01, and *∗∗∗* indicates *p* < 0.001 when compared with control.

**Table 7:** Effect of *D. candenatensis* on Hole Cross test in mice (mean ± SEM, *n* = 5).

Group	Number of holes crossed by the mice				
	0 min	30 min	60 min	90 min	120 min
Negative control	6.5 ± 0.64	6.0 ± 0.41	5.75 ± 0.48	5.75 ± 0.48	5.5 ± 0.64
Positive control	6.75 ± 0.85	4.75 ± 0.75	2.25 ± 0.48^*∗∗*^	1.25 ± 0.48^*∗∗∗*^	1.75 ± 0.48^*∗∗*^
Test I (100 mg/kg)	6.0 ± 0.41	5.5 ± 0.87	4.75 ± 0.48	4.50 ± 0.64	5.0 ± 0.71
Test II (250 mg/kg)	6.25 ± 0.62	5.50 ± 0.96	4.75 ± 1.03	4.25 ± 0.48	4.0 ± 1.08
Test III (500 mg/kg)	6.0 ± 1.08	5.75 ± 0.48	3.5 ± 0.64	3.0 ± 0.71^*∗*^	3.25 ± 1.18

*∗* indicates *p* < 0.05, *∗∗* indicates *p* < 0.01, and *∗∗∗* indicates *p* < 0.001 when compared with control.

**Table 8:** Effect of *D. candenatensis* on Hole Board Test in mice (mean ± SEM, *n* = 5).

Group	Number of head dips by the mice				
	0 min	30 min	60 min	90 min	120 min
Negative control	30.0 ± 3.29	32.25 ± 2.28	32.0 ± 3.34	32.75 ± 2.06	32.5 ± 2.22
Positive control	29.75 ± 1.11	21.0 ± 1.82^*∗*^	13.25 ± 0.85^*∗∗∗*^	8.75 ± 0.85^*∗∗∗*^	12.0 ± 1.08^*∗∗∗*^
Test I (100 mg/kg)	30.25 ± 4.30	30.75 ± 4.03	28.0 ± 4.06	25.0 ± 3.02	24.0 ± 1.9
Test II (250 mg/kg)	33.75 ± 2.32	25.25 ± 3.12	22.5 ± 2.53	19.0 ± 1.96^*∗∗*^	18.5 ± 1.04^*∗∗*^
Test III (500 mg/kg)	28.25 ± 3.70	25.5 ± 1.55	17.5 ± 1.71^*∗∗∗*^	15.0 ± 2.85^*∗∗∗*^	15.0 ± 1.58^*∗∗∗*^

*∗* indicates  *p* < 0.05, *∗∗* indicates *p* < 0.01, and *∗∗∗* indicates *p* < 0.001 when compared with control.

## Supplementary Materials

Supplementary Materials Figure  2: Supplementary raw data of acetic acid induced writhing (analgesic activity) test in mice. Table  3: Supplementary raw data of acetic acid induced writhing (analgesic activity) test in mice. Table  4: Supplementary raw data of anti-inflammatory activity test.
