# Supplementary material for: In Vivo and In Vitro Evaluation of Pharmacological Potentials of Secondary Bioactive Metabolites of Dalbergia candenatensis Leaves
Source: Evid Based Complement Alternat Med. 2017 Dec 26;2017:5034827. doi: 10.1155/2017/5034827 (PMC5758950; doi:10.1155/2017/5034827)
Supplement: Supplementary Materials — Figure 2: Supplementary raw data of acetic acid induced writhing (analgesic activity) test in mice. Table 3: Supplementary raw data of acetic acid induced writhing (analgesic activity) test in mice. Table 4: Supplementary raw data of anti-inflammatory activity test. [file 5034827.f1.pdf]

Supplementary file for manuscript 5034827 titled "Evaluation of secondary bioactive metabolites pharmacological potentials from *Dalbergia candenatensis* leaves"

Figure - 2 Supplementary raw data of acetic acid induced writhing (Analgesic activity) test in mice

Vincrstin sulphate (LC_{50})

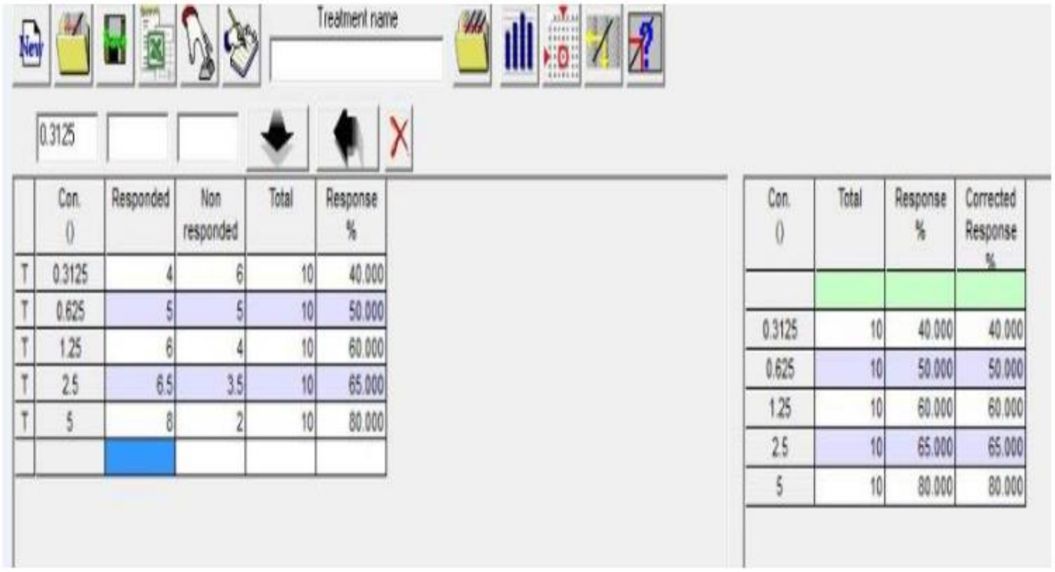

	Con. ()	Responded	Non responded	Total	Response %
T	0.3125	4	6	10	40.000
T	0.625	5	5	10	50.000
T	1.25	6	4	10	60.000
T	2.5	6.5	3.5	10	65.000
T	5	8	2	10	80.000

Con. ()	Total	Response %	Corrected Response %
0.3125	10	40.000	40.000
0.625	10	50.000	50.000
1.25	10	60.000	60.000
2.5	10	65.000	65.000
5	10	80.000	80.000

***Dalbergia candenatensis* (LC_{50})**

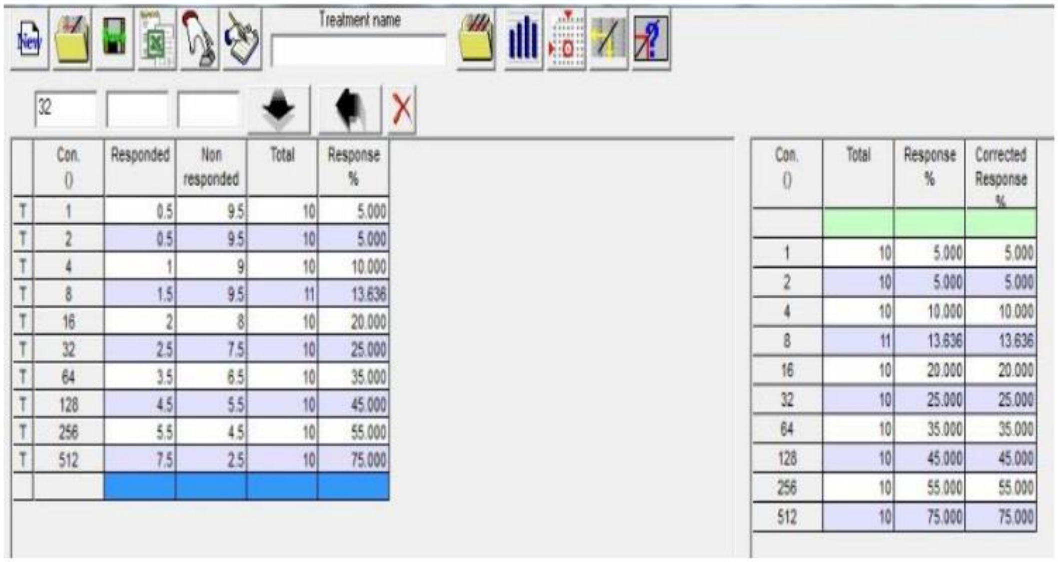

	Con. ()	Responded	Non responded	Total	Response %
T	1	0.5	9.5	10	5.000
T	2	0.5	9.5	10	5.000
T	4	1	9	10	10.000
T	8	1.5	9.5	11	13.636
T	16	2	8	10	20.000
T	32	2.5	7.5	10	25.000
T	64	3.5	6.5	10	35.000
T	128	4.5	5.5	10	45.000
T	256	5.5	4.5	10	55.000
T	512	7.5	2.5	10	75.000

Con. ()	Total	Response %	Corrected Response %
1	10	5.000	5.000
2	10	5.000	5.000
4	10	10.000	10.000
8	11	13.636	13.636
16	10	20.000	20.000
32	10	25.000	25.000
64	10	35.000	35.000
128	10	45.000	45.000
256	10	55.000	55.000
512	10	75.000	75.000

Table- 3 Supplementary raw data of acetic acid induced writhing (Analgesic activity) test in mice

S.L. No.	Animal Groups	Dose	Weight of mice (g)	No. of writhing	Mean writhing	% writhing	% inhibition of writhing
1	Control (Tween-80 water -1% w/v)	10 ml/kg	21	21	19.60±1.81	100	0
			26	18			
			23	17			
			24	26			
			27	16			
2	Diclofenac sodium	25 mg/kg	26	3	4.60±0.68*	23.47	76.53
			29	5			
			30	4			
			27	7			
			25	4			
3	Extract I	100 mg/kg	24	14	15.00±0.71*	76.53	23.47
			28	13			
			27	17			
			21	16			
			22	15			
4	Extract II	250 mg/kg	23	10	11.60±1.08*	59.18	40.82
			24	15			
			24	13			
			21	11			
			29	09			
5	Extract III	500 mg/kg	27	13	8.4±1.33*	42.86	57.14
			24	08			
			29	07			
			24	05			
			26	09			

Data was presented as mean ± SEM, SEM= Standard Error Mean, Here, * indicates $p<0.05$.

Table – 4 Supplementary raw data of anti-inflammatory activity test

Groups	Dose (mg/kg)	Difference in paw diameter					
		**1 hr**	**2 hr**	**3 hr**	**4 hr**	**24 hr**	**48 hr**
Control (1% v/v Tween-80 water)	10 ml/kg	1.10	1.24	1.29	1.31	1.28	1.25
		1.11	1.24	1.28	1.36	1.33	1.27
		1.09	1.26	1.27	1.32	1.32	1.25
		1.12	1.27	1.28	1.34	1.29	1.24
		1.08	1.25	1.27	1.30	1.28	1.30
Indomethacin	10 mg/kg	0.81	0.84	0.47	0.51	0.60	0.62
		0.82	0.85	0.45	0.49	0.56	0.59
		0.82	0.86	0.43	0.53	0.55	0.60
		0.80	0.85	0.47	0.50	0.57	0.65
		0.84	0.86	0.44	0.52	0.59	0.61
Extract I	100 mg/kg	1.06	1.17	1.07	1.10	1.07	1.06
		1.05	1.19	1.01	1.12	1.09	1.10
		1.07	1.20	1.05	1.09	1.05	1.09
		1.03	1.16	1.03	1.13	1.10	1.07
		1.05	1.18	1.04	1.11	1.08	1.06
Extract II	250 mg/kg	1.01	1.10	0.92	0.99	0.98	0.93
		1.02	1.11	0.94	0.96	0.95	0.97
		1.00	1.12	0.89	1.00	0.99	0.99
		1.03	1.13	0.93	0.97	0.95	0.94
		1.02	1.09	0.91	0.98	0.97	0.96
Extract II	500 mg/kg	0.96	1.08	0.80	0.84	0.79	0.83
		0.97	1.10	0.79	0.86	0.83	0.84
		0.95	1.07	0.81	0.82	0.86	0.81
		0.99	1.07	0.82	0.87	0.84	0.82
		0.98	1.06	0.81	0.81	0.82	0.79
